# Application of pseudocontinuous arterial spin labeling perfusion imaging in children with autism spectrum disorders

**DOI:** 10.3389/fnins.2022.1045585

**Published:** 2022-11-08

**Authors:** Fang Ye, Lei Du, Bing Liu, Xinying Gao, Aocai Yang, Die Liu, Yue Chen, Kuan Lv, Pengfei Xu, Yuanmei Chen, Jing Liu, Lipeng Zhang, Shijun Li, Amir Shmuel, Qi Zhang, Guolin Ma

**Affiliations:** ^1^Department of Pediatrics, China-Japan Friendship Hospital, Beijing, China; ^2^Department of Radiology, China-Japan Friendship Hospital, Beijing, China; ^3^Key Laboratory of Carcinogenesis and Translational Research, Ministry of Education, Department of Radiology, Peking University, Cancer Hospital and Institute, Beijing, China; ^4^Graduate School of Peking Union Medical College, Chinese Academy of Medical Sciences, Beijing, China; ^5^Peking University China-Japan Friendship School of Clinical Medicine, Beijing, China; ^6^Department of Radiology, First Medical Center of Chinese PLA General Hospital, Beijing, China; ^7^McConnell Brain Imaging Centre, Montreal Neurological Institute, McGill University, Montreal, QC, Canada; ^8^Department of Neurology and Neurosurgery, McGill University, Montreal, QC, Canada

**Keywords:** cerebral blood flow, arterial spin labeling, autism spectrum disorders, magnetic resonance imaging, children

## Abstract

**Introduction:**

Pseudocontinuous Arterial Spin Labeling (pCASL) perfusion imaging allows non-invasive quantification of regional cerebral blood flow (CBF) as part of a multimodal magnetic resonance imaging (MRI) protocol. This study aimed to compare regional CBF in autism spectrum disorders (ASD) individuals with their age-matched typically developing (TD) children using pCASL perfusion imaging.

**Materials and methods:**

This cross-sectional study enrolled 17 individuals with ASD and 13 TD children. All participants underwent pCASL examination on a 3.0 T MRI scanner. Children in two groups were assessed for clinical characteristics and developmental profiles using Autism Behavior Checklist (ABC) and Gesell development diagnosis scale (GDDS), respectively. We compared CBF in different cerebral regions of ASD and TD children. We also assessed the association between CBF and clinical characteristics/developmental profile.

**Results:**

Compared with TD children, individuals with ASD demonstrated a reduction in CBF in the left frontal lobe, the bilateral parietal lobes, and the bilateral temporal lobes. Within the ASD group, CBF was significantly higher in the right parietal lobe than in the left side. Correlation analysis of behavior characteristics and CBF in different regions showed a positive correlation between body and object domain scores on the ABC and CBF of the bilateral occipital lobes, and separately, between language domain scores and CBF of the left frontal lobe. The score of the social and self-help domain was negatively correlated with the CBF of the left frontal lobe, the left parietal lobe, and the left temporal lobe.

**Conclusion:**

Cerebral blood flow was found to be negatively correlated with scores in the social and self-help domain, and positively correlated with those in the body and object domain, indicating that CBF values are a potential MRI-based biomarker of disease severity in ASD patients. The findings may provide novel insight into the pathophysiological mechanisms of ASD.

## Introduction

Autism spectrum disorders (ASD) is a group of disabilities characterized by impairments in social interaction and communication, as well as restricted, repetitive, and stereotyped patterns of behavior, activities, or interests (Pardo and Eberhart, 2007; Sztainberg and Zoghbi, 2016). In recent decades, ASD has become one of the most relevant neurodevelopmental disorders in children and, therefore, an urgent public health problem. This group of disorders is considered a large unmet medical need and a heavy burden on individuals with ASD, their families, and society worldwide (Buescher et al., 2014). To date, the prevalence of ASD is one in 44 children in the United States (Kogan et al., 2018).

As a behavioral disease, ASD is considered to be highly correlated with brain dysfunction. At present, the pathology and etiology of ASD remain uncertain, and its clinical diagnosis is mainly based on medical observation and clinical symptom evaluation of the patient by a certified specialist. Disorders that are characterized by atypical social functioning, such as ASD, are known to correlate with impaired neural network function. The establishment of quantitative objective biomarkers (e.g., cerebral perfusion and cerebral function) for ASD would aid in the auxiliary diagnosis of ASD. In recent decades, neuroimaging studies have provided new insights into the neurobiological underpinnings of behavioral impairments associated with ASD (Ecker et al., 2015; Mori et al., 2015; Walsh et al., 2021).

Among the above-mentioned neuroimaging studies, multimodal magnetic resonance imaging (MRI) techniques have been used to understand the pathophysiological characteristics of ASD in recent years, including structural MRI (sMRI), functional MRI (fMRI), ASL, etc. sMRI examination has been widely used to investigate brain morphology. Two major types of sMRI have been used in studies: geometric features and volumetric features (Ozgen et al., 2013). However, sMRI cannot determine how brain regions communicate and how information is integrated across networks (Chen et al., 2011). To further identify metabolic disorders of the brain instead of structural abnormalities, fMRI has been used in several ASD studies (Chen et al., 2016; Abraham et al., 2017; Sherkatghanad et al., 2019). This technique estimates neuronal activity based on the blood oxygen level dependent (BOLD) signal (Yin et al., 2021) that reflects fluctuations in brain blood oxygenation levels. However, the BOLD signal is an indirect measure of neuronal activity. BOLD signal results from the combination of cerebral blood flow (CBF), cerebral blood volume, and cerebral metabolic rate of oxygen consumption (Chen et al., 2015). To overcome this limitation and utilize a more direct and quantitative measure of brain metabolism, ASL has been increasingly adopted as a novel functional technique.

Arterial Spin Labeling is based on magnetically labeled endogenous water in the blood and using it as a tracer (Ohnishi et al., 2000; Zheng et al., 2019; Wang et al., 2022). This approach enables the quantification of CBF without radiation exposure (Hernandez-Garcia et al., 2019). The ASL method offers several advantages: it is safe, non-invasive, non-radioactive, easy to acquire, and reproducible. Its high spatial resolution, accuracy, and capacity to quantitatively demonstrate perfusion abnormalities make ASL a reliable physiological marker of neural activity (Chen et al., 2015; Ivanov et al., 2017; Li et al., 2020). It has been widely used in areas of neurodevelopmental disorders such as periventricular leukomalacia, ischemic stroke, and sickle-cell-related cerebral ischemia (Smith et al., 2019). It also has the potential to become a good biomarker for ASD studies (Mori et al., 2015).

Arterial Spin Labeling can be divided into three types according to different labeling methods: pulsed ASL (PASL), continuous ASL (CASL), and pseudocontinuous ASL (pCASL). pCASL is a hybrid approach that simulates CASL using many short pulses to achieve continuous labeling for better compensation of adverse magnetization transfer effects (Boudes et al., 2014). It could improve the sensitivity and temporal signal-to-noise ratio (SNR) of ASL. This technique allows both reliable CBF measurement and functional connectivity analysis of perfusion image series because the potential contributions of the blood oxygenation level-dependent are minimized (Jann et al., 2015; Zhao et al., 2022).

In recent years, several studies have focused on CBF changes in ASD individuals. These studies indicated decreased blood flow (hypoperfusion) in the cerebrum regions of ASD individuals compared with neurotypical children (Hashimoto et al., 2000; Ohnishi et al., 2000; Yerys et al., 2018). Hashimoto et al. (2000) identified a decrease in CBF in both the frontal and temporal lobes in autistic brains using the CBF single-photon emission computed tomography (SPECT) method. Ohnishi et al. (2000) found hypoperfusion in the insula, superior temporal gyrus (STG), and prefrontal area in young individuals with ASD. Yerys et al. (2018) demonstrated a reduction in CBF in regions critical to social perception and cognition in children with ASD.

Previous studies have primarily focused on the comparison of CBF between ASD and typically developed individuals. To the best of our knowledge, the relationship between changes in CBF and the clinical characteristics and developmental profile of ASD remains unknown. The present study was designed to detect regional CBF changes in ASD individuals using the three-dimensional pCASL (3D–pCASL) technique and to further explore the correlation between ASD characteristics and CBF changes. Our results provide novel insight to support the investigation of the pathophysiological mechanisms of ASD.

## Materials and methods

### Participants

Thirty-two children [19 in ASD; 13 in typically developing (TD)] between the ages of 3 and 8 were invited to participate in this study. The inclusion criteria for the children in the ASD group are as follows: (1) 3–8 years old; (2) meet the diagnostic criteria of the Diagnostic and Statistical Manual of Mental Disorders–the 5th edition (DSM-V) checklist for autism (Ben-Ari et al., 2022); (3) have an Autism Behavior Checklist (ABC) score ≥53; (4) right-handedness; (5) no other diseases that may affect brain function and structure, with no abnormalities on routine head MRI; and (6) no history of medical treatment or intervention. To be enrolled in the ASD group, the child was first screened by a pediatrician using the ABC. Then, the diagnosis of the child was performed by a pediatric expert according to DSM-V (Ben-Ari et al., 2022). Subsequently, their intellectual functioning was evaluated by an experienced developmental-behavioral pediatrician based on the Gesell development diagnosis scale (GDDS).

The inclusion criteria for children in the control group are as follows: (1) 3–8 years old; (2) normally developed; (3) have an ABC score ≤ 53, have a DQ ≥ 75 or IQ ≥ 70; (4) right-handedness; and (5) no other diseases that may affect brain function and structure, with no abnormalities on routine head MRI. To be enrolled in the TD group, participants were first screened by a pediatrician using the ABC and then the GDDS (for children 3–6 years old) or Raven’s Standard Progressive Matrices (RPM) (for children over 6 years old). And the exclusion criteria for both groups are as follows: (1) have abnormal brain lesions; (2) do not cooperate with sedation; (3) suffer from epilepsy; (4) without informed consent obtained from the parents; and (5) parents reported any known genetic, neurological, or psychiatric disorder. Two eligible children withdrew from the study prior to the MRI examination. Ultimately, there were 17 individuals with ASD and 13 TD children enrolled in this study.

### Assessment tool

#### Autism behavior checklist

The ABC is a 57-item questionnaire used to assess behavioral problems in ASD children (Volkmar et al., 1988). Previous domestic studies have shown that the ABC has good reliability and validity and is one of the most commonly used autism symptom assessment tools in China (Zhu et al., 2017). In this study, we used the ABC as a tool to assess the severity of autism symptoms. The total score directly correlated with the severity of behavioral symptoms. The ABC is divided into five subscales, including problems related to sensory (sensation and perception; 10 items), relating (relation and connection; 14 items), body and object use (physical activity and rigid use of objects; 9 items), language (communication and interaction; 13 items), and social and self-help (adaptability and self-care; 11 items) domains. All items were rated as “yes” (scored from 1 to 4 with different symptoms) or “no” (scored as 0 without symptom) for each question during the assessment. The score ranges from 0 to 158, with score greater than 53 indicating ASD. In this study, we utilized this test for participant screening and assessing autistic symptoms.

#### Gesell development diagnosis scale

The GDDS is one of the gold standards for neurodevelopment assessment and one of the most commonly used tools for assessing neurodevelopment in children (Wei et al., 2021). The GDDS can comprehensively and continuously reflect individual neurodevelopment and is widely used in clinical and scientific research in China. It uses direct observation to evaluate a child’s cognitive, language, motor, and social-emotional responses in the domains of gross motor, fine motor, adaptive behavior, language and social behavior. The development quotient (DQ) of each domain was calculated for each participant. The DQ less than 75 indicates developmental retardation. In this study, we utilized this test for participant screening if the child was younger than 6.

#### Raven’s standard progressive matrices

The RPM is a commonly used test of intelligence quotient (IQ) that contains multiple choice questions pertaining to abstract reasoning (David and Skilbeck, 1984). In each test question in the RPM, the child is asked to identify the missing item that completes a pattern. The IQ less than 70 indicates mental retardation. In this study, we utilized this test for participant screening if the child was over 6 years old.

### Study procedures

All participants completed three data collection sessions. The first session was an evaluation to confirm that individuals had ASD or were neurotypical. Then, the GDDS was conducted on the ASD individuals for DQ evaluation. All participants then completed an MRI scan. The study was approved by the Institutional Review Board of China–Japan Friendship Hospital. Informed consent was obtained from the caregivers of all the participants after the purpose and risks of the study had been fully explained. As all the participants could not cooperate with the MRI examination, they were administered with chloral hydrate 50 mg/kg orally, with maximum dose of 1 g.

### Magnetic resonance imaging scanning

A 3-Tesla MRI with 8-channel head coils was used for scanning (Discovery MR 750 scanner, GE Medical Systems, USA). pCASL with one post-labeling delay (PLD) was performed in the study. All perfusion and reference images were obtained with a 3-dimensional stack-of spirals rapid acquisition with refocused echoes imaging sequence (Stivaros et al., 2018). The imaging parameters were as follows: repetition time, 4,632 ms; PLD, 1,500 ms; echo time, 10.5 ms; flip angle, 90 degrees; field of view, 240 mm × 240 mm; bandwidth = 6250 Hz/pixel; slice thickness, 4 mm; number of slices, 36; and total scan time, 3 min 24 s.

### Data processing

**FIGURE 1:**
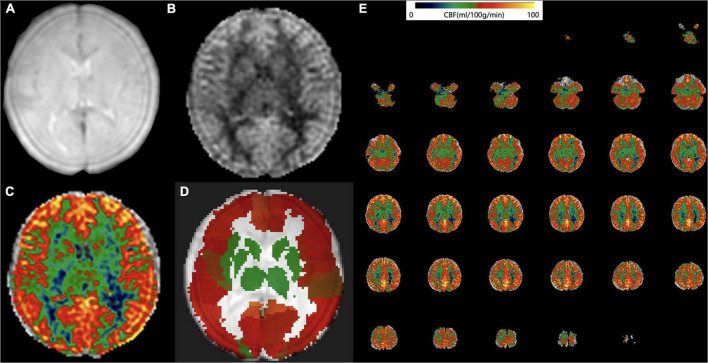
Arterial spin labeling (ASL) data processing workflow. Convert the **(A)** 3D ASL proton density images and **(B)** perfusion weighted images into the cerebral blood flow (CBF) map of cerebral perfusion for each participant. **(C)** The CBF map was normalized to the Montreal Neurological Institute (MNI) space and then the Automated anatomical labelling (AAL) atlas was overlaid. **(D)** The average CBF value of each brain region from AAL atlas was obtained. **(E)** An example CBF map of a four-year old male ASD patient calculated by Cereflow software.

Arterial Spin Labeling data were processed using the CereFlow software (An–Image Technology Co, China). The procedures were as follows:

(1) The 3-dimensional ASL perfusion-weighted images and proton density images (generated from the GE MR scanner) were converted into the CBF map of cerebral perfusion for each participant based on the simplified one compartment model (St Lawrence and Wang, 2005).
(2) The CBF map was normalized to the Montreal Neurological Institute (MNI) space by using intensity-based image registration. In this image registration method, each participant’s CBF map served as the motion image and would be transformed to match the MNI152 brain template that acted as the fixed image.
(3) Then, the Automated anatomical labeling (AAL) atlas was overlaid onto the normalized CBF map.
(4) The average CBF value of different regions was obtained (Figure 1).

### Statistical analysis

Analyses were performed using the SPSS 22.0 software (Chicago, IL, United States). The Shapiro–Wilk test and Levene’s test were used to assess data normality and homogeneity, respectively. Student’s *t*-test was used for variables with a normal distribution and equal variance, while the Kolmogorov–Smirnov test was used for those with a skewed distribution or unequal variance. CBF within the bilateral hemispheres was compared using a paired *t*-test. Pearson and Spearman correlation analyses were conducted between CBF and scores of the GDDS and ABC assessment tools for normally or skewed distributed variables, respectively. Partial correlation analysis was utilized to control for the effect of possible confounders of sex and age. Sensitivity analysis was conducted to evaluate whether the outliers affect the outcomes by comparing the results before and after eliminating the outliers. To control for multiple comparisons (Breznik et al., 2020), the Benjamini-Hochberg false discovery rate (FDR) procedure was used, and corrected *q*-values less than 0.05 were designated as statistically significant.

## Results

### Characteristics of participants

The demographic profile and clinical characteristics of all 17 ASD individuals and 13 TD children are summarized in Table 1. The two groups were balanced in age, but the percentage of male participants was higher in the ASD group. However, we performed a subgroup analysis to further compare CBF between male and female participants, and no significant difference was observed (Supplementary Table 1).

**TABLE 1:** Demographic and clinical characteristics of participants.

Characteristic	ASD (*n* = 17)		TD (*n* = 13)		*Z-*value^c^	*P*-value
	Median	IQR	Median	IQR		
**Demographic profile**						
Male sex	15^a^	88.2^b^	5^a^	38.5^b^	6.126^d^	**0.013**
Age (years)	4.0	2.0	4.5	4.0	1.093	0.183
**Clinical characteristic**						
**Scores on ABC subscales**						
Total	68.0	17.0	10.0	9.5	4.627	**<0.001**
Sensory	10.0	3.5	2.0	3.0	4.656	**<0.001**
Relating	15.0	10.5	2.0	3.5	4.558	**<0.001**
Body and object use	5.0	9.5	0.0	1.5	3.130	**0.002**
Language	21.0	6.5	1.0	3.0	4.641	**<0.001**
Social and self-help skills	14.0	5.5	3.0	2.5	4.280	**<0.001**
**Developmental profile^e^**						
**DQ of GDDS subscales**						
Total	68.6	16.0	100.0	4.3	3.961	**<0.001**
Cognition	69.0	20.0	100.0	10.8	3.965	**<0.001**
Gross motor	84.0	21.5	105.5	16.5	3.971	**<0.001**
Minor motor	73.0	14.5	99.0	7.8	3.822	**<0.001**
Language	53.0	12.5	100.0	11.3	3.967	**<0.001**
Personal-Social behavior	68.0	20.0	101.5	5.3	3.908	**<0.001**

ASD, autism spectrum disorders; TD, typically developing; ABC, autism behavior checklist; GDDS, Gesell development diagnosis scale; DQ, development quotient; IQ, intelligence quotient; IQR, interquartile range. *^a^*n of male participants. ^b^% of male participants. *^c^*Z-value of Wilcoxon rank sum test. *^d^*Continuity correction value of Chi-Square. ^e^5 children in TD group were tested for IQ, the median of which was 99.0, and IQR was 5.0. Bold values mean statistically significant.

### Comparison of cerebral blood flow in different regions between the two groups

**FIGURE 2:**
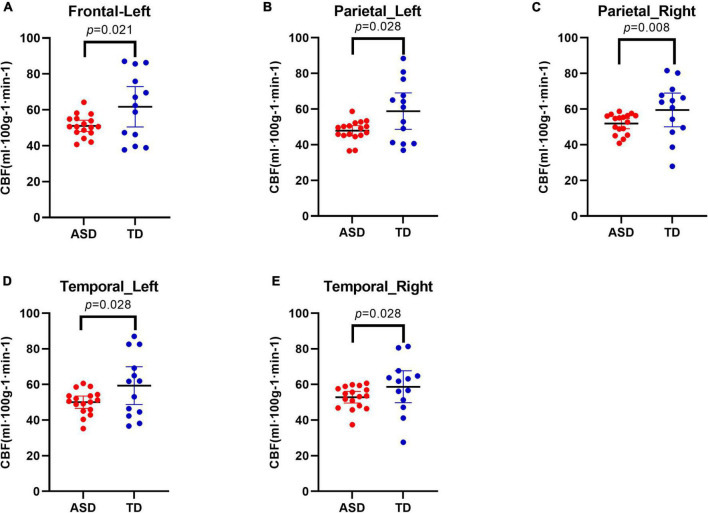
Decreased cerebral blood flow (CBF) in different regions in Autism Spectrum Disorders (ASD) group compared with typically developing (TD) group. Results showed decreased CBF in the left frontal lobe **(A)**, in the bilateral parietal lobes **(B,C)**, and in the bilateral temporal lobes **(D,E)**.

Compared with TD individuals, ASD individuals exhibited significant reductions in CBF in the left frontal lobe (*q*-value = 0.045), bilateral parietal lobes (both *q*-values = 0.045), and bilateral temporal lobes (both *q*-values = 0.045) (Figure 2).

### Comparison of cerebral blood flow in different regions between bilateral hemispheres

Within each group, we compared the CBF between homologous regions in the two hemispheres (Table 2). In the ASD group, the CBF in the parietal lobe of the left hemisphere was lower than that in the right hemisphere (*p* = 0.032). No significant difference was observed in the TD group.

**TABLE 2:** Comparison of CBF between homologous regions in the two hemispheres.

	Left hemisphere	Right hemisphere	*t*-value	*q*-value^a^
**ASD (*n* = 17)**				
Frontal lobe	51.1 ± 6.0	51.7 ± 6.2	0.502	0.787
Parietal lobe	47.9 ± 5.6	51.9 ± 5.6	3.371	**0.032**
Temporal lobe	50.0 ± 6.7	52.8 ± 6.3	2.433	0.080
Occipital lobe	54.2 ± 7.7	56.0 ± 7.2	2.382	0.080
**TD (*n* = 13)**				
Frontal lobe	61.7 ± 18.6	59.7 ± 15.8	0.892	0.780
Parietal lobe	58.8 ± 16.9	59.5 ± 15.6	0.309	0.787
Temporal lobe	59.3 ± 17.5	58.7 ± 14.9	0.276	0.787
Occipital lobe	59.2 ± 14.9	59.6 ± 14.4	0.601	0.787

CBF, cerebral blood flow; ASD, autism spectrum disorders; TD, typically developing. *^a^*Benjamini-Hochberg FDR procedure for multiple comparison correction was used and *q*-values were reported, *q*-value < 0.05 were considered significant. Bold values mean statistically significant.

### Correlation analysis between clinical characteristics and cerebral blood flow of different regions within the two groups

**FIGURE 3:**
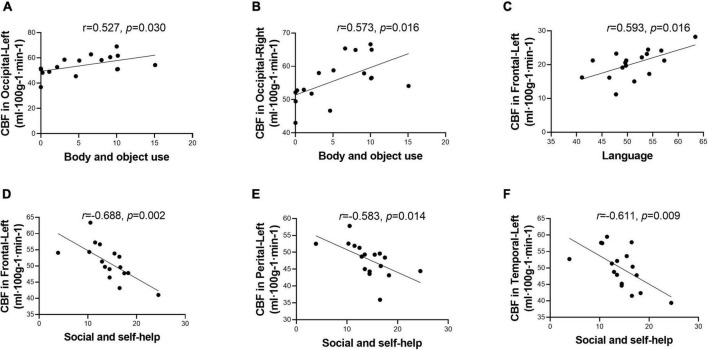
Score of Autism Behavior Checklist (ABC) domains correlated with cerebral blood flow (CBF) in different regions. Score of body and object use was positively correlated with CBF in the bilateral occipital lobes **(A,B)**. Score of language was positively correlated with CBF in the left frontal lobe **(C)**. Score of social and self-help was negatively correlated with CBF in the left hemisphere, including frontal lobe, perietal lobe, and temporal lobe **(D–F)**. While after multiple comparisons correction, the correlation was not statistically signicant.

Within the ASD group, the body and object domain score on the ABC was positively correlated with the CBF of the bilateral occipital lobes (Figures 3A,B). The language domain score on the ABC was positively correlated with the CBF of the left frontal lobe (Figure 3C). In addition, the social and self-help domain score was negatively correlated with the CBF of the left frontal lobe (Figure 3D), left parietal lobe (Figure 3E), and left temporal lobe (Figure 3F).

However, after correcting for multiple comparisons, all the correlations were not statistically significant. Besides, no statistically significant correlation was observed in the TD group (Supplementary Table 2).

### Correlation analysis between developmental indicators and cerebral blood flow of different regions within the two groups

The correlation between the DQ of GDDS and the CBF of different regions was analyzed, but no statistically significant correlation was observed (Supplementary Table 3).

### Sensitivity analysis of outliers within autism spectrum disorders group

**FIGURE 4:**
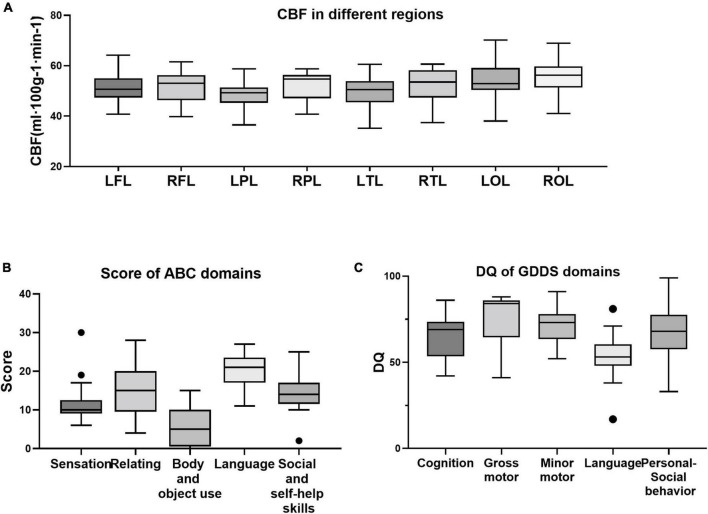
Distribution of all variables of cerebral blood flow (CBF) **(A)**, clinical characteristics **(B)**, and developmental indicators **(C)**. Within ASD group, outliers were detected in the sensation domain score on the Autism Behavior Checklist (ABC) **(B)**, the social and self-help domain score on the ABC **(B)**, and the language domain DQ on the Gesell development diagnosis scale (GDDS) **(C)**.

Figure 4 presents the distributions of all the variables obtained from the ASD group. Outliers were detected in three variables, including the sensation domain score on the ABC, the social and self-help domain score on the ABC, and the language domain DQ on the GDDS. The results of the sensitivity analysis indicated that all statistically significant findings prevailed after the exclusion of outliers (Supplementary Tables 4, 5).

## Discussion

Using the 3D-pCASL technique, the current study investigated the patterns of CBF changes in ASD individuals. To the best of our knowledge, this study is the first to explore CBF changes and their relationships with clinical characteristics. ASD pathogenesis is not yet completely understood. The present study moves us closer to identifying biologically based marker that relates ASD to a basic social perception deficit. This study also highlights the value of ASL neuroimaging in the field of ASD.

One of the major findings is the different CBF characteristics in individuals with ASD compared with typically developed controls. In line with previous findings, we identified CBF decreases in the left frontal lobe (Hashimoto et al., 2000), the bilateral parietal lobes (Yerys et al., 2018), and the bilateral temporal lobes (Yerys et al., 2018; Tang et al., 2022).

First, CBF, which is a variable that reflects neurovascular coupling, is regulated by local neuronal activity and metabolism (Iadecola, 2004). Local CBF reductions affect ATP synthesis, diminishing (Na^+^-K^+^) ATPase activity and the capacity of neurons to generate action potentials (Kalaria, 2010). The decreased activity of (Na^+^-K^+^) ATPase further contributes to the accumulation of glutamate (Zlokovic, 2011). Glutamate is the most prevalent excitatory neurotransmitter. Based on data from human patients and animal models, excess glutamate leads to an elevation in cortical excitability, which has been proposed to be a fundamental neurobiological characteristic of ASD (Bozzi et al., 2018; Horder et al., 2018; Antoine et al., 2019; Sohal and Rubenstein, 2019). Local circuit hyperexcitability could contribute to aberrations in brain connectivity in autism. Alternatively, excess glutamate activates glutamate receptors, leading to the production of nitric oxide (NO) (Reynell and Harris, 2013; Raju et al., 2015). As a signaling molecule in the central nervous system (CNS), NO is one of the most essential messenger molecules (Son et al., 1996; Raju et al., 2015). NO-mediated signaling contributes to cerebrovascular and synaptic transmission coupling in the CNS (Son et al., 1996). Studies have confirmed that NO leads to the S-nitrosylation (SNO) of many proteins and receptors through various pathways (Amal et al., 2020). SNO targets a wide range of prominent intracellular proteins, which leads to alterations in specific signaling pathways. These alterations may cause deficits in synapses, neurons, and behavior (Tripathi et al., 2020).

Second, the abnormal patterns of CBF in essential regions of the cerebrum would affect corresponding functions. The frontal lobes support high-level cognition; they are extremely important for the development of social behavior. Recent studies have implied that the medial prefrontal cortex (mPFC), the so-called “social brain” (Hiser and Koenigs, 2018), is involved in social behaviors (Hazlett et al., 2017). The “social brain” region is involved in computing and associating reward value with various types of stimulation. This association is relevant to “decreased social motivation,” an explanatory theory of autism (Chevallier et al., 2012). In addition, studies have found that the orbitofrontal cortex, another region of the frontal lobes, computes individual preferences and social hierarchies (Azzi et al., 2012). A deficit in this cortical region was considered to underlie social function disorders, which is a typical defect in ASD (Jonker et al., 2015). Moreover, the posterior cingulate gyrus located in the frontal lobe is an important part of emotional circuits (Miao et al., 2011). Two studies (Lee et al., 2013; Sophia et al., 2013) have reported dysfunction in this area in ASD patients.

Joint attention is an early indicator and a key deficit in autism (Osterling and Dawson, 1994). This deficit was proven to be associated with executive function, which was described as a set of cognitive processes including planning, working memory, impulse control, inhibition, and shifting set, as well as the initiation and monitoring of action (Gallagher and Frith, 2003). Structural studies of the autistic brain identified delayed postnatal maturation of the frontal lobes (Zilbovicius et al., 1995) and structural abnormalities in the orbitofrontal cortex (Salmond et al., 2003). In line with previous results, we found hypoperfusion in the left frontal lobe, and a negative correlation between CBF and scores in the social and self-help domains in our study. The results indicated functional abnormalities or abnormal social overture in this region. Further studies should explore executive function and characteristics in neuroimaging studies in ASD.

Interestingly, the current study showed that the ABC scores of social and self-help skills were negatively correlated with CBF in the left frontal lobe. Social and self-help skills were thought to be based on executive functions, which are defined as a set of interrelated cognitive processes required for complex goal-directed activity. The frontal lobe is highly evolved and regulate complex behaviors, which include complex cognitive functions such as planning complex cognitive behaviors, personality expression, decision-making, and regulating social behavior. Thus, hypofrontality may contribute to the clinical symptoms in ASD (Diedrichsen et al., 2019). In addition, distinct cognitive and behavioral processes are mediated by different regions of the frontal lobe. Further studies should focus on the correlation between symptoms in ASD individuals and CBF changes within subregions of the cerebrum, particularly in the frontal lobe. Different CBF patterns in this region may be one of the characteristics of ASD individuals.

Within the temporal lobes, we identified hypoperfusion and a negative association with social and self-help domain scores. This finding was consistent with significantly reduced activation in the left STG and the superior temporal sulcus (STS) in autistic children with Fragile X Syndrome (Philip et al., 2012). In addition, one of the hypotheses of ASD indicates a primary factor of abnormal processing of the information conveyed by behavior (Kaiser et al., 2012). This hypothesis suggests that understanding and using gaze or emotional expressions requires specific neural mechanisms, and the STG, STS, and the temporal–parietal junction have been selectively associated with the processing of body and face movement (Annaz et al., 2010). The atypical processing of bodily movement and gaze might be a vital factor in ASD.

Within the bilateral parietal lobes, our study revealed lower perfusion in ASD individuals and a negative correlation between CBF and the ABC score of the social and self-help domains. This finding is consistent with the consensus from numerous studies: patients with lesions in the parietal lobe exhibit difficulty with self-care, such as dressing and washing (Glasser et al., 2016). This difficulty may be related to the sensory and linguistic functions and specific structures in the parietal lobes. The parietal lobes are related to sensory and linguistic functions (Johnson et al., 2011). Located in the parietal lobes, the angular gyrus is one of the most important centers of visual language. Through the function of the angular gyrus, various forms of information generate integrated language activities, since the left angular gyrus connects the auditory, visual, and somatosensory cortices (Hofmann et al., 2008). Liang et al. (2021) found that the number of functional connectivity neural circuits in the left angular gyrus was abnormally decreased in the ASD group. This decrease may be related to lower perfusion in this area, as shown in our study. Both decreased functional connectivity and lower perfusion indicate cognitive dysfunction in ASD patients and the disconnection between what they see and what they hear (Liang et al., 2021).

The lack of correlation between the developmental performance and reduction of CBF suggests that the difficulty experienced by ASD individuals in daily life is not necessarily correlated with their DQ, which was in line with a previous study (Mori et al., 2015).

Different reviews noted that the language-relevant cortex includes Broca’s area in the inferior frontal gyrus, Wernicke’s area in the STG, parts of the middle temporal gyrus, and the inferior parietal and angular gyrus in the parietal lobe (Friederici, 2002; Hickok and Poeppel, 2007). This finding is in accordance with our results that the ABC language scores were positively correlated with CBF in the left frontal lobe, where Broca’s area is located.

Our results showed that body and object use scores were positively correlated with CBF in the bilateral occipital lobes. The key function of the occipital lobe, where the visual cortex is located, is processing visual information (Van Essen et al., 2018). Visuospatial processing and distance perception, which are also related to the function of the occipital lobe, may play a key part in the process of “body and object use.” These results suggested the involvement of the occipital lobe in the performance of ASD individuals.

Derived from MRI using ASL, CBF changes are thought to reflect regional changes in neural activity over time (Detre et al., 2012). Furthermore, CBF provides quantitative absolute measures associated with regional brain function. Therefore, it may provide a more reliable marker of trait-like effects than measures obtained through contrasting conditions, such as BOLD fMRI which is typically used in task-based studies. The advantages of ASL position it as an optimal non-invasive biological marker of ASD.

Several limitations of our study should be acknowledged. First, we enrolled a relatively small sample due to the strict inclusion and exclusion criteria, which may affect the statistical reliability of our results. Further studies should focus on establishing the diagnostic value of CBF in ASD individuals with larger sample sizes among different populations. Second, the sex ratio was skewed in this study. We compared male and female participants in subgroup analyses. The results showed no difference in any CBF or behavioral data. Moreover, partial correlation analysis was conducted to control for the effect of possible confounders of gender and age. In a future study, we will enroll more female ASD individuals to minimize the effect of sex, and subgroup analysis will be conducted to explore the difference in CBF between male and female ASD individuals. Third, the CBF data analyzed in the current study were collected from each lobe of the cerebrum, and further studies should focus on more specific cortical regions in ASD individuals.

## Data availability statement

The raw data supporting the conclusions of this article will be made available by the authors, without undue reservation.

## Ethics statement

The studies involving human participants were reviewed and approved by the Ethics Committee of China–Japan Friendship Hospital. Written informed consent to participate in this study was provided by the participants’ legal guardian/next of kin.

## Author contributions

FY, LD, and BL acquired and analyzed the ASL data and drafted the manuscript. DL, XYG, JL, YMC, and LPZ searched and managed the literature. KL, YC, and ACY did the MRI scanning. PFX, SJL, and AS revised the manuscript. GLM and QZ designed the study and revised the manuscript. All authors contributed to the article and approved the submitted version.

## References

[B1] Abraham A. Milham M. P. Di Martino A. Craddock R. C. Samaras D. Thirion B. (2017). ‘Deriving reproducible biomarkers from multi-site resting-state data: An Autism-based example’. *Neuroimage* 147 736–745. 10.1016/j.neuroimage.2016.10.045 27865923

[B2] Amal H. Barak B. Bhat V. Gong G. Joughin B. A. Wang X. (2020). ‘Shank3 mutation in a mouse model of autism leads to changes in the S-nitroso-proteome and affects key proteins involved in vesicle release and synaptic function’. *Mol. Psychiatry* 25 1835–1848. 10.1038/s41380-018-0113-6 29988084 PMC6614015

[B3] Annaz D. Remington A. Milne E. Coleman M. Campbell R. Thomas M. S. (2010). ‘Development of motion processing in children with autism’. *Dev. Sci.* 13 826–838. 10.1111/j.1467-7687.2009.00939.x 20977554

[B4] Antoine M. W. Langberg T. Schnepel P. Feldman D. E. (2019). ‘Increased excitation-inhibition ratio stabilizes synapse and circuit excitability in four autism mouse models’. *Neuron* 101 648–661.e4. 30679017 10.1016/j.neuron.2018.12.026 PMC6733271

[B5] Azzi J. C. Sirigu A. Duhamel J. R. (2012). ‘Modulation of value representation by social context in the primate orbitofrontal cortex’. *Proc. Natl. Acad. Sci. U.S.A.* 109 2126–2131. 10.1073/pnas.1111715109 22308343 PMC3277550

[B6] Ben-Ari Y. Caly H. Rabiei H. Lemonnier É (2022). ‘Early prognostic of ASD: A challenge’. *Med. Sci.* 38 431–437. 10.1051/medsci/2022054 35608465

[B7] Boudes E. Gilbert G. Leppert I. R. Tan X. Pike G. B. Saint-Martin C. (2014). ‘Measurement of brain perfusion in newborns: Pulsed arterial spin labeling (PASL) versus pseudo-continuous arterial spin labeling (pCASL)’. *Neuroimage Clin.* 6 126–133. 10.1016/j.nicl.2014.08.010 25379424 PMC4215516

[B8] Bozzi Y. Provenzano G. Casarosa S. (2018). ‘Neurobiological bases of autism-epilepsy comorbidity: A focus on excitation/inhibition imbalance’. *Eur. J. Neurosci.* 47 534–548. 10.1111/ejn.13595 28452083

[B9] Breznik E. Malmberg F. Kullberg J. Ahlström H. Strand R. (2020). ‘Multiple comparison correction methods for whole-body magnetic resonance imaging’. *J. Med. Imaging* 7:014005. 10.1117/1.Jmi.7.1.014005 PMC7047011 32206683

[B10] Buescher A. V. Cidav Z. Knapp M. Mandell D. S. (2014). ‘Costs of autism spectrum disorders in the United Kingdom and the United States’. *JAMA Pediatr.* 168 721–728. 10.1001/jamapediatrics.2014.210 24911948

[B11] Chen H. Duan X. Liu F. Lu F. Ma X. Zhang Y. (2016). ‘Multivariate classification of autism spectrum disorder using frequency-specific resting-state functional connectivity–a multi-center study’. *Prog. Neuropsychopharmacol. Biol. Psychiatry* 64 1–9. 10.1016/j.pnpbp.2015.06.014 26148789

[B12] Chen J. J. Jann K. Wang D. J. (2015). ‘Characterizing resting-state brain function using arterial spin labeling’. *Brain Connect.* 5 527–542. 10.1089/brain.2015.0344 26106930 PMC4652156

[B13] Chen R. Jiao Y. Herskovits E. H. (2011). ‘Structural MRI in autism spectrum disorder’. *Pediatr. Res.* 69:63R–8R. 10.1203/PDR.0b013e318212c2b3 21289538 PMC3081653

[B14] Chevallier C. Kohls G. Troiani V. Brodkin E. S. Schultz R. T. (2012). ‘The social motivation theory of autism’. *Trends Cogn. Sci.* 16 231–239. 10.1016/j.tics.2012.02.007 22425667 PMC3329932

[B15] David R. M. Skilbeck C. E. (1984). ‘Raven IQ and language recovery following stroke’. *J. Clin. Neuropsychol.* 6 302–308. 10.1080/01688638408401220 6206094

[B16] Detre J. A. Rao H. Wang D. J. Chen Y. F. Wang Z. (2012). ‘Applications of arterial spin labeled MRI in the brain’. *J. Magn. Reson. Imaging* 35 1026–1037. 10.1002/jmri.23581 22246782 PMC3326188

[B17] Diedrichsen J. King M. Hernandez-Castillo C. Sereno M. Ivry R. B. (2019). ‘Universal transform or multiple functionality? understanding the contribution of the human cerebellum across task domains’. *Neuron* 102 918–928. 10.1016/j.neuron.2019.04.021 31170400 PMC6686189

[B18] Ecker C. Bookheimer S. Y. Murphy D. G. (2015). ‘Neuroimaging in autism spectrum disorder: Brain structure and function across the lifespan’. *Lancet Neurol.* 14 1121–1134. 10.1016/s1474-4422(15)00050-2 25891007

[B19] Friederici A. D. (2002). ‘Towards a neural basis of auditory sentence processing’. *Trends Cogn. Sci.* 6 78–84. 10.1016/s1364-6613(00)01839-8 15866191

[B20] Gallagher H. L. Frith C. D. (2003). ‘Functional imaging of ’theory of mind”. *Trends Cogn. Sci.* 7 77–83. 10.1016/s1364-6613(02)00025-6 12584026

[B21] Glasser M. F. Coalson T. S. Robinson E. C. Hacker C. D. Harwell J. Yacoub E. (2016). ‘A multi-modal parcellation of human cerebral cortex’. *Nature* 536 171–178. 10.1038/nature18933 27437579 PMC4990127

[B22] Hashimoto T. Sasaki M. Fukumizu M. Hanaoka S. Sugai K. Matsuda H. (2000). ‘Single-photon emission computed tomography of the brain in autism: Effect of the developmental level’. *Pediatr. Neurol.* 23 416–420. 10.1016/s0887-8994(00)00224-1 11118797

[B23] Hazlett H. C. Gu H. Munsell B. C. Kim S. H. Styner M. Wolff J. J. (2017). ‘Early brain development in infants at high risk for autism spectrum disorder’. *Nature* 542 348–351. 10.1038/nature21369 28202961 PMC5336143

[B24] Hernandez-Garcia L. Lahiri A. Schollenberger J. (2019). ‘Recent progress in ASL’. *Neuroimage* 187 3–16. 10.1016/j.neuroimage.2017.12.095 29305164 PMC6030511

[B25] Hickok G. Poeppel D. (2007). ‘The cortical organization of speech processing’. *Nat. Rev. Neurosci.* 8 393–402. 10.1038/nrn2113 17431404

[B26] Hiser J. Koenigs M. (2018). ‘The multifaceted role of the ventromedial prefrontal cortex in emotion, decision making, social cognition, and psychopathology’. *Biol. Psychiatry* 83 638–647. 10.1016/j.biopsych.2017.10.030 29275839 PMC5862740

[B27] Hofmann M. J. Herrmann M. J. Dan I. Obrig H. Conrad M. Kuchinke L. (2008). Differential activation of frontal and parietal regions during visual word recognition: An optical topography study. *NeuroImage* 40, 1340–1349. 10.1016/j.neuroimage.2007.12.037 18262438

[B28] Horder J. Petrinovic M. M. Mendez M. A. Bruns A. Takumi T. Spooren W. (2018). ‘Glutamate and GABA in autism spectrum disorder-a translational magnetic resonance spectroscopy study in man and rodent models’. *Transl. Psychiatry* 8:106. 10.1038/s41398-018-0155-1 29802263 PMC5970172

[B29] Iadecola C. (2004). ‘Neurovascular regulation in the normal brain and in Alzheimer’s disease’. *Nat. Rev. Neurosci.* 5 347–360. 10.1038/nrn1387 15100718

[B30] Ivanov D. Gardumi A. Haast R. A. M. Pfeuffer J. Poser B. A. Uludağ K. (2017). ‘Comparison of 3T and 7T ASL techniques for concurrent functional perfusion and BOLD studies’. *Neuroimage* 156 363–376. 10.1016/j.neuroimage.2017.05.038 28528845

[B31] Jann K. Gee D. G. Kilroy E. Schwab S. Smith R. X. Cannon T. D. (2015). ‘Functional connectivity in BOLD and CBF data: Similarity and reliability of resting brain networks’. *Neuroimage* 106 111–122. 10.1016/j.neuroimage.2014.11.028 25463468 PMC4285775

[B32] Johnson R. Simon E. J. Henkell H. Zhu J. (2011). The role of episodic memory in controlled evaluative judgments about attitudes: An event-related potential study. *Neuropsychologia* 49, 945–960. 10.1016/j.neuropsychologia.2011.01.028 21262245

[B33] Jonker F. A. Jonker C. Scheltens P. Scherder E. J. (2015). ‘The role of the orbitofrontal cortex in cognition and behavior’. *Rev. Neurosci.* 26 1–11. 10.1515/revneuro-2014-0043 25252749

[B34] Kaiser M. D. Shiffrar M. Pelphrey K. A. (2012). ‘Socially tuned: Brain responses differentiating human and animal motion’. *Soc. Neurosci.* 7 301–310. 10.1080/17470919.2011.614003 21943047

[B35] Kalaria R. N. (2010). ‘Vascular basis for brain degeneration: Faltering controls and risk factors for dementia’. *Nutr. Rev.* 68:S74–S87. 10.1111/j.1753-4887.2010.00352.x 21091952 PMC3058404

[B36] Kogan M. D. Vladutiu C. J. Schieve L. A. Ghandour R. M. Blumberg S. J. Zablotsky B. (2018). ‘The prevalence of parent-reported autism spectrum disorder among US children’. *Pediatrics* 142:e20174161. 10.1542/peds.2017-4161 30478241 PMC6317762

[B37] Lee M. H. Smyser C. D. Shimony J. S. (2013). ‘Resting-state fMRI: A review of methods and clinical applications’. *AJNR Am. J. Neuroradiol.* 34 1866–1872. 10.3174/ajnr.A3263 22936095 PMC4035703

[B38] Li X. Zhao P. Qiu X. Ding H. Lv H. Yang Z. (2020). ‘Lateralization effects on cerebral blood flow in patients with unilateral pulsatile tinnitus measured with arterial spin labeling’. *Front. Hum. Neurosci.* 14:591260. 10.3389/fnhum.2020.591260 33281587 PMC7705237

[B39] Liang D. Xia S. Zhang X. Zhang W. (2021). ‘Analysis of brain functional connectivity neural circuits in children with autism based on persistent homology’. *Front. Hum. Neurosci.* 15:745671. 10.3389/fnhum.2021.745671 34588970 PMC8473898

[B40] Miao X. Wu X. Li R. Chen K. Yao L. (2011). ‘Altered connectivity pattern of hubs in default-mode network with Alzheimer’s disease: An granger causality modeling approach’. *PLoS One* 6:e25546. 10.1371/journal.pone.0025546 22022410 PMC3191142

[B41] Mori K. Toda Y. Ito H. Mori T. Mori K. Goji A. (2015). ‘Neuroimaging in autism spectrum disorders: 1H-MRS and NIRS study’. *J. Med. Investig.* 62 29–36. 10.2152/jmi.62.29 25817280

[B42] Ohnishi T. Matsuda H. Hashimoto T. Kunihiro T. Nishikawa M. Uema T. (2000). ‘Abnormal regional cerebral blood flow in childhood autism’. *Brain* 123 1838–1844. 10.1093/brain/123.9.1838 10960047

[B43] Osterling J. Dawson G. (1994). ‘Early recognition of children with autism: A study of first birthday home videotapes’. *J. Autism Dev. Disord.* 24 247–257. 10.1007/bf02172225 8050980

[B44] Ozgen H. Hellemann G. S. de Jonge M. V. Beemer F. A. van Engeland H. (2013). ‘Predictive value of morphological features in patients with autism versus normal controls’. *J. Autism Dev. Disord.* 43 147–155. 10.1007/s10803-012-1554-4 22669539 PMC3536966

[B45] Pardo C. A. Eberhart C. G. (2007). ‘The neurobiology of autism’. *Brain Pathol.* 17 434–447. 10.1111/j.1750-3639.2007.00102.x 17919129 PMC8095519

[B46] Philip R. C. Dauvermann M. R. Whalley H. C. Baynham K. Lawrie S. M. Stanfield A. C. (2012). ‘A systematic review and meta-analysis of the fMRI investigation of autism spectrum disorders’. *Neurosci. Biobehav. Rev.* 36 901–942. 10.1016/j.neubiorev.2011.10.008 22101112

[B47] Raju K. Doulias P. T. Evans P. Krizman E. N. Jackson J. G. Horyn O. (2015). ‘Regulation of brain glutamate metabolism by nitric oxide and S-nitrosylation’. *Sci. Signal.* 8:ra68. 10.1126/scisignal.aaa4312 26152695 PMC4746709

[B48] Reynell C. Harris J. J. (2013). ‘The BOLD signal and neurovascular coupling in autism’. *Dev. Cogn. Neurosci.* 6 72–79. 10.1016/j.dcn.2013.07.003 23917518 PMC3989023

[B49] Salmond C. H. de Haan M. Friston K. J. Gadian D. G. Vargha-Khadem F. (2003). ‘Investigating individual differences in brain abnormalities in autism’. *Philos. Trans. R. Soc. Lond. B Biol. Sci.* 358 405–413. 10.1098/rstb.2002.1210 12639337 PMC1693120

[B50] Sherkatghanad Z. Akhondzadeh M. Salari S. Zomorodi-Moghadam M. Abdar M. Acharya U. R. (2019). ‘Automated detection of autism spectrum disorder using a convolutional neural network’. *Front. Neurosci.* 13:1325. 10.3389/fnins.2019.01325 32009868 PMC6971220

[B51] Smith L. G. F. Milliron E. Ho M. L. Hu H. H. Rusin J. Leonard J. (2019). ‘Advanced neuroimaging in traumatic brain injury: An overview’. *Neurosurg. Focus* 47:E17. 10.3171/2019.9.Focus19652 32364704

[B52] Sohal V. S. Rubenstein J. L. R. (2019). ‘Excitation-inhibition balance as a framework for investigating mechanisms in neuropsychiatric disorders’. *Mol. Psychiatry* 24 1248–1257. 10.1038/s41380-019-0426-0 31089192 PMC6742424

[B53] Son H. Hawkins R. D. Martin K. Kiebler M. Huang P. L. Fishman M. C. (1996). ‘Long-term potentiation is reduced in mice that are doubly mutant in endothelial and neuronal nitric oxide synthase’. *Cell* 87 1015–1023. 10.1016/s0092-8674(00)81796-1 8978606

[B54] Sophia M. Daniel K. Samson A. C. Valerie K. Janusch B. Michel G. (2013). Convergent findings of altered functional and structural brain connectivity in individuals with high functioning autism: a multimodal MRI study. *PLoS One* 8:e67329. 10.1371/journal.pone.0067329 23825652 PMC3688993

[B55] St Lawrence K. S. Wang J. (2005). Effects of the apparent transverse relaxation time on cerebral blood flow measurements obtained by arterial spin labeling. *Magn. Reson. Med.* 53, 425–433. 10.1002/mrm.20364 15678532

[B56] Stivaros S. Garg S. Tziraki M. Cai Y. Thomas O. Mellor J. (2018). ‘Randomised controlled trial of simvastatin treatment for autism in young children with neurofibromatosis type 1 (SANTA)’. *Mol. Autism* 9:12. 10.1186/s13229-018-0190-z 29484149 PMC5824534

[B57] Sztainberg Y. Zoghbi H. Y. (2016). ‘Lessons learned from studying syndromic autism spectrum disorders’. *Nat. Neurosci.* 19 1408–1417. 10.1038/nn.4420 27786181

[B58] Tang S. Liu X. Ran Q. Nie L. Wu L. Pan Z. (2022). ‘Application of three-dimensional pseudocontinuous arterial spin labeling perfusion imaging in the brains of children with autism’. *Front. Neurol.* 13:851430. 10.3389/fneur.2022.851430 35280268 PMC8905523

[B59] Tripathi M. K. Kartawy M. Amal H. (2020). ‘The role of nitric oxide in brain disorders: Autism spectrum disorder and other psychiatric, neurological, and neurodegenerative disorders’. *Redox Biol.* 34:101567. 10.1016/j.redox.2020.101567 32464501 PMC7256645

[B60] Van Essen D. C. Donahue C. J. Glasser M. F. (2018). ‘Development and evolution of cerebral and cerebellar cortex’. *Brain Behav. Evol.* 91 158–169. 10.1159/000489943 30099464 PMC6097530

[B61] Volkmar F. R. Cicchetti D. V. Dykens E. Sparrow S. S. Leckman J. F. Cohen D. J. (1988). ‘An evaluation of the autism behavior checklist’. *J. Autism Dev. Disord.* 18 81–97. 10.1007/bf02211820 3372461

[B62] Walsh M. J. M. Wallace G. L. Gallegos S. M. Braden B. B. (2021). ‘Brain-based sex differences in autism spectrum disorder across the lifespan: A systematic review of structural MRI, fMRI, and DTI findings’. *Neuroimage Clin.* 31:102719. 10.1016/j.nicl.2021.102719 34153690 PMC8233229

[B63] Wang X. H. Liu X. F. Ao M. Wang T. He J. Gu Y. W. (2022). ‘Cerebral perfusion patterns of anxiety state in patients with pulmonary nodules: A study of cerebral blood flow based on arterial spin labeling’. *Front. Neurosci.* 16:912665. 10.3389/fnins.2022.912665 35615271 PMC9125149

[B64] Wei X. Hu J. Yang L. Gao M. Li L. Ding N. (2021). ‘Bidirectional association of neurodevelopment with growth: A prospective cohort study’. *BMC Pediatr.* 21:203. 10.1186/s12887-021-02655-7 33910531 PMC8080371

[B65] Yerys B. E. Herrington J. D. Bartley G. K. Liu H. S. Detre J. A. Schultz R. T. (2018). ‘Arterial spin labeling provides a reliable neurobiological marker of autism spectrum disorder’. *J. Neurodev. Disord.* 10:32. 10.1186/s11689-018-9250-0 30541425 PMC6292037

[B66] Yin W. Mostafa S. Wu F. X. (2021). ‘Diagnosis of Autism Spectrum Disorder Based on Functional Brain Networks with Deep Learning’. *J. Comput. Biol.* 28 146–165. 10.1089/cmb.2020.0252 33074746

[B67] Zhao M. Y. Woodward A. Fan A. P. Chen K. T. Yu Y. Chen D. Y. (2022). ‘Reproducibility of cerebrovascular reactivity measurements: A systematic review of neuroimaging techniques()’. *J. Cereb. Blood Flow Metab.* 42 700–717. 34806918 10.1177/0271678X211056702 PMC9254040

[B68] Zheng W. Ren S. Zhang H. Liu M. Zhang Q. Chen Z. (2019). ‘Spatial Patterns of Decreased Cerebral Blood Flow and Functional Connectivity in Multiple System Atrophy (Cerebellar-Type): A Combined Arterial Spin Labeling Perfusion and Resting State Functional Magnetic Resonance Imaging Study’. *Front. Neurosci.* 13:777. 10.3389/fnins.2019.00777 31417345 PMC6685442

[B69] Zhu J. Guo M. Yang T. Lai X. Lei Y. He M. (2017). ‘Association between behavioral problems and gastrointestinal disorders among children with autism spectrum disorder’. *Chin. J. Pediatr.* 55 905–910. 10.3760/cma.j.issn.0578-1310.2017.12.007 29262469

[B70] Zilbovicius M. Garreau B. Samson Y. Remy P. Barthélémy C. Syrota A. (1995). ‘Delayed maturation of the frontal cortex in childhood autism’. *Am. J. Psychiatry* 152 248–252. 10.1176/ajp.152.2.248 7840359

[B71] Zlokovic B. V. (2011). ‘Neurovascular pathways to neurodegeneration in Alzheimer’s disease and other disorders’. *Nat. Rev. Neurosci.* 12 723–738. 10.1038/nrn3114 22048062 PMC4036520

