# Supplementary material for: Application of pseudocontinuous arterial spin labeling perfusion imaging in children with autism spectrum disorders
Source: Front Neurosci. 2022 Nov 8;16:1045585. doi: 10.3389/fnins.2022.1045585 (PMC9680558; doi:10.3389/fnins.2022.1045585)
Supplement: Supplementary file 1 [file Data_Sheet_1.docx]

**TABLE S1** Comparison of CBF between male and female participants within two groups.

| CBF  (ml/100 g/min) | ASD (Median(IQR)) | | *p*-value | TD (Median(IQR)) | | *p*-value |
| --- | --- | --- | --- | --- | --- | --- |
|  | Male | Female |  | Male | Female |  |
| Left frontal lobe | 50.60(6.40) | 43.95(6.50) | 0.121 | 46.20(27.55) | 68.25(23.48) | 0.141 |
| Right frontal lobe | 53.00(10.30) | 48.20(14.40) | 0.121 | 48.80(31.70) | 63.40(13.10) | 0.895 |
| Left parietal lobe | 49.60(6.91) | 41.20(9.40) | 0.310 | 41.20(24.35) | 64.60(25.47) | 0.413 |
| Right parietal lobe | 54.70(7.50) | 50.85(15.70) | 0.310 | 49.50(33.70) | 65.50(13.48) | 0.770 |
| Left temporal lobe | 51.20(8.40) | 42.85(15.30) | 0.121 | 44.50(25.05) | 63.40(24.05) | 0.697 |
| Right temporal lob | 53.50(9.50) | 48.40(22.00) | 0.121 | 51.30(27.50) | 63.40(8.98) | 0.770 |
| Left occipital lobe | 52.90(8.10) | 50.15(17.30) | 0.310 | 48.60(23.35) | 63.80(18.73) | 0.895 |
| Right occipital lobe | 56.26(8.30) | 50.35(18.70) | 0.491 | 49.60(28.00) | 61.05(14.95) | 0.770 |
| Score of ABC | 68.00(16.00) | 81.50(23.0) | 0.635 | 11.00(7.00) | 6.50(12.00) | 0.413 |
| DQ of GDDS | 68.60(15.80) | 70.20(9.60) | 0.925 | 99.60(10.8) | 100.40(4.70) | 0.990 |

*CBF, cerebral blood flow; ASD, autism spectrum disorders; IQR, interquartile range; TD, typically developing; ABC, Autism Behavior Checklist; DQ, development quotient; GDDS, Gesell development diagnosis scale.*

**TABLE S2** Partial correlation coefficient between the score of ABC and CBF in different regions in TD children

| r (correlation coefficient) ^a^ | LFL | RFL | LPL | RPL | LTL | RTL | LOL | ROL |
| --- | --- | --- | --- | --- | --- | --- | --- | --- |
| ABC total | 0.185 | 0.019 | 0.168 | 0.099 | 0.127 | 0.000 | 0.160 | 0.128 |
| Sensory | 0.207 | 0.051 | 0.201 | 0.130 | 0.122 | 0.054 | 0.136 | 0.108 |
| Relating | 0.090 | 0.076 | 0.006 | 0.079 | 0.042 | 0.020 | 0.073 | 0.031 |
| Body and Object use | 0.149 | -0.135 | 0.196 | 0.054 | 0.078 | -0.007 | 0.101 | 0.085 |
| Language | 0.097 | -0.085 | 0.051 | -0.017 | 0.077 | -0.074 | 0.097 | 0.055 |
| Social and Self-help skills | 0.092 | 0.064 | 0.164 | 0.014 | 0.070 | -0.017 | 0.075 | 0.064 |

*ABC, Autism Behavior Checklist; CBF, cerebral blood flow; TD, typically developing; LFL, left frontal lobe; RFL, right frontal lobe; LPL, left parietal lobe; RPL, right parietal lobe; LTL, left temporal lobe; RTL, right temporal lobe; LOL, left occipital lobe; ROL, right occipital lobe.*

^a^ *all p value of r > 0.05.*

**TABLE S3** Partial correlation coefficients between DQ of GDDS and CBF in different regions for children with ASD and TD children

| r (correlation coefficient) ^a^ | LFL | | RFL | | LPL | | RPL | | LTL | | RTL | | LOL | | ROL | |
| --- | --- | --- | --- | --- | --- | --- | --- | --- | --- | --- | --- | --- | --- | --- | --- | --- |
|  | ASD | TD | ASD | TD | ASD | TD | ASD | TD | ASD | TD | ASD | TD | ASD | TD | ASD | TD |
| DQ of GDDS subscales |  |  |  |  |  |  |  |  |  |  |  |  |  |  |  |  |
| Cognition | -0.418 | 0.265 | -0.064 | 0.193 | -0.292 | 0.181 | 0.117 | 0.277 | -0.311 | 0.265 | 0.167 | 0.096 | -0.185 | 0.313 | -0.023 | 0.315 |
| Gross motor | -0.376 | -0.419 | 0.065 | -0.359 | -0.271 | -0.611 | 0.186 | -0.539 | -0.222 | -0.419 | 0.102 | -0.539 | -0.241 | -0.515 | 0.065 | -0.488 |
| Minor motor | -0.043 | -0.037 | 0.016 | -0.086 | 0.170 | -0.209 | 0.1.78 | -0.037 | 0.025 | -0.037 | 0.177 | -0.209 | -0.018 | -0.037 | 0.032 | 0.000 |
| Language | -0.271 | -0.473 | 0.050 | -0.655 | -0.141 | 0.473 | 0.382 | -0.618 | -0.286 | -0.473 | 0.101 | -0.473 | -0.206 | -0.546 | -0.020 | -0.549 |
| Personal-Social behavior | -0.179 | 0.267 | 0.291 | 0.364 | -0.103 | 0.024 | 0.298 | 0.182 | -0.011 | 0.267 | 0.338 | 0.206 | 0.064 | 0.133 | 0.191 | 0.110 |

*DQ, development quotient; GDDS, Gesell development diagnosis scale; CBF, cerebral blood flow; ASD, autism spectrum disorders; TD, typically developing; LFL, left frontal lobe; RFL, right frontal lobe; LPL, left parietal lobe; RPL, right parietal lobe; LTL, left temporal lobe; RTL, right temporal lobe; LOL, left occipital lobe; ROL, right occipital lobe.*

*^a^ all p value of r > 0.05.*

**TABLE S4** Sensitivity analysis of partial correlation analysis between the score of sensation domain of ABC and CBF in different regions

|  | Before eliminating the outliers  (n=17) | | | After eliminating the outliers  (n=15) | | |
| --- | --- | --- | --- | --- | --- | --- |
|  | *r* | *p value* | *q value ^a^* | *r* | *p value* | *q value* |
| Left frontal lobe | -0.280 | 0.312 | 0.312 | -0.589 | 0.134 | 0.414 |
| Right frontal lobe | -0.379 | 0.164 | 0.236 | -0.386 | 0.193 | 0.414 |
| Left parietal lobe | -0.318 | 0.248 | 0.283 | -0.375 | 0.207 | 0.414 |
| Right parietal lobe | -0.434 | 0.106 | 0.236 | -0.168 | 0.582 | 0.582 |
| Left temporal lobe | -0.410 | 0.129 | 0.236 | -0.686 | 0.110 | 0.414 |
| Right temporal lobe | -0.499 | 0.059 | 0.236 | -0.222 | 0.465 | 0.546 |
| Left occipital lobe | -0.368 | 0.177 | 0.236 | -0.280 | 0.354 | 0.546 |
| Right occipital lobe | -0.413 | 0.126 | 0.236 | -0.216 | 0.478 | 0.546 |

*^a^ Benjamini-Hochberg FDR procedure for multiple comparison correction was used and q-values were reported, q value <0.05 was considered significant.*

**TABLE S5** Sensitivity analysis of partial correlation analysis between the scores of social and self-help domain of ABC and CBF in different regions

|  | Before eliminating the outlier  (n=17) | | | After eliminating the outlier  (n=16) | | |
| --- | --- | --- | --- | --- | --- | --- |
|  | *r* | *p-value* | *q value ^a^* | *r* | *p-value* | *q value* |
| Left frontal lobe | -0.688 | 0.002 | 0.016 | -0.775 | 0.001 | 0.008 |
| Right frontal lobe | -0.489 | 0.064 | 0.128 | -0.420 | 0.134 | 0.213 |
| Left parietal lobe | -0.583 | 0.014 | 0.037 | -0.571 | 0.033 | 0.037 |
| Right parietal lobe | -0.264 | 0.341 | 0.341 | -0.207 | 0.477 | 0.213 |
| Left temporal lobe | -0.611 | 0.009 | 0.036 | -0.698 | 0.006 | 0.024 |
| Right temporal lob | -0.268 | 0.335 | 0.341 | -0.380 | 0.180 | 0.213 |
| Left occipital lobe | -0.444 | 0.097 | 0.155 | -0.452 | 0.104 | 0.208 |
| Right occipital lobe | -0.339 | 0.217 | 0.289 | -0.375 | 0.186 | 0.213 |

*^a^ Benjamini-Hochberg FDR procedure for multiple comparison correction was used and q-values were reported, q value <0.05 was considered significant.*
